# Can molecular hydrogen supplementation enhance physical performance in healthy adults? A systematic review and meta-analysis

**DOI:** 10.3389/fnut.2024.1387657

**Published:** 2024-06-05

**Authors:** Kaixiang Zhou, Zhangyuting Shang, Chaoqun Yuan, Zhenxiang Guo, Yubo Wang, Dapeng Bao, Junhong Zhou

**Affiliations:** ^1^College of Physical Education and Health Science, Chongqing Normal University, Chongqing, China; ^2^College of Physical Education and Health Management, Chongqing University of Education, Chongqing, China; ^3^College of Sports and Health, Chengdu University of Traditional Chinese Medicine, Chengdu, China; ^4^Sports Coaching College, Beijing Sport University, Beijing, China; ^5^China Institute of Sport and Health Science, Beijing Sport University, Beijing, China; ^6^Hebrew SeniorLife Hinda and Arthur Marcus Institute for Aging Research, Harvard Medical School, Boston, MA, United States

**Keywords:** molecular hydrogen, physical performance, aerobic endurance, maximum oxygen uptake, maximal anaerobic test, muscle strength, countermovement jump

## Abstract

**Background:**

Physical exertion during exercise often leads to increased oxidative stress and inflammatory responses, significantly affecting physical performance. Current strategies to mitigate these effects are limited by their effectiveness and potential side effects. Molecular hydrogen (H₂) has gained attention for its antioxidant and anti-inflammatory properties. Studies have suggested that H_2_ supplementation contributes to antioxidant potential and anti-fatigue during exercise, but the variance in the observations and study protocols is presented across those studies.

**Objective:**

This systematic review and meta-analysis aimed to comprehensively characterize the effects of H₂ supplementation on physical performance (i.e., endurance, muscular strength, and explosive power), providing knowledge that can inform strategies using H_2_ for enhancing physical performance.

**Methods:**

We conducted a literature search of six databases (PubMed, Web of Science, Medline, Sport-Discus, Embase, and PsycINFO) according to the PRISMA guidelines. The data were extracted from the included studies and converted into the standardized mean difference (SMD). After that, we performed random-effects meta-analyses and used the *I*^2^ statistic to evaluate heterogeneity. The Grading of Recommendations Assessment, Development, and Evaluation (GRADE) was used to assess the quality of the evidence obtained from this meta-analysis.

**Results:**

In total, 27 publications consisting of 597 participants were included. The search finally included aerobic endurance, anaerobic endurance, muscular strength, lower limb explosive power, rating of perceived exertion (RPE), blood lactate (BLA), and average heart rate (HR_avg_) in the effect size (ES) synthesis. The ES of H_2_ on aerobic endurance, including V̇O_2max_ (SMD = 0.09, *p* = 0.394; *I*^2^ = 0%) and aerobic endurance exercise (SMD = 0.04, *p* = 0.687; *I*^2^ = 0%), were not significant and trivial; the ES of H_2_ on 30 s maximal anaerobic endurance (SMD = 0.19, *p* = 0.239; *I*^2^ = 0%) was not significant and trivial; the ES of H_2_ on muscular strength (SMD = 0.19, *p* = 0.265; *I*^2^ = 0%) was not significant and trivial; but the ES of H_2_ on lower limb explosive power (SMD = 0.30, *p* = 0.018; *I*^2^ = 0%) was significant and small. In addition, H_2_ reduces RPE (SMD = −0.37, *p* = 0.009; *I*^2^ = 58.0%) and BLA (SMD = −0.37, *p* = 0.001; *I*^2^ = 22.0%) during exercise, but not HR_avg_ (SMD = −0.27, *p* = 0.094; *I*^2^ = 0%).

**Conclusion:**

These findings suggest that H_2_ supplementation is favorable in healthy adults to improve lower limb explosive power, alleviate fatigue, and boost BLA clearance, but may not be effectively improving aerobic and anaerobic endurance and muscular strength. Future studies with more rigorous designs are thus needed to examine and confirm the effects of H_2_ on these important functionalities in humans.

**Systematic review registration:**

http://www.crd.york.ac.uk/PROSPERO.

## 1 Introduction

Physical performance, including endurance, muscle strength, and explosive power, is the cornerstone of achievement in sports for non-athletic populations or athletes (1, 2). It not only contributes to improving athletes’ competitive performance on the field but also provides motivation for healthy adults to participate in sports (3–5). Oxidative stress occurs when the oxygen metabolism is produced and accumulated, eventually going beyond oxidation-resist ability (6, 7). Studies have shown that physical activity of various intensities alters the levels of various oxidative biomarkers (8, 9). However, physical exercise, especially with moderate- to high-intensity exertion, could lead to excessive oxidative stress, which may negatively impact redox homeostasis, worsen fatigue, and ultimately reduce physical performance (10–13). Therefore, efforts have been put into exploring potential antioxidant approaches, which can thus help develop appropriate strategies to enhance physical performance (13–15).

Molecular hydrogen (H_2_) is a promising antioxidant that selectively reduces hydroxyl radicals (·OH) and peroxynitrite (ONOO-) in cells without leading to a reduction of other reactive substances such as superoxide (O_2_-), hydrogen peroxide (H_2_O_2_), and nitric oxide (NO) (16–18). Studies have shown that H_2_ molecular, which can be delivered via different forms (i.e., H_2_ gas and water, and intravenous H_2_-saline), can penetrate cell membranes and diffuse rapidly into organelles (e.g., mitochondria) (19), thus enhancing mitochondria functional performance (e.g., respiration and enzyme activity) and promoting ATP production or lactate oxidation (20, 21). More recently, human studies have emerged to explore the potential benefits of using H_2_ for physical performance in healthy adults and showed great promise of the H_2_-based intervention to improve physical performance (22–25). However, the observations and protocol design across these studies on the effects of H_2_ on physical performance were inconsistent. For example, some studies have observed that H_2_-rich water (HRW) supplementation before exercise could effectively increase maximal oxygen uptake (V̇O_2max_), anaerobic endurance, muscle strength, and lower limb explosive power in healthy adults (26–28), but other studies have shown contradictory findings (29–31). These inconsistencies may arise from the variance in participant characteristics, the protocol of H_2_ administration, and types of exercise across studies. Only one previous review by Kawamura et al. (32) summarized the observations from only six studies and suggested that the validity of the observations from that literature should be examined and confirmed due to the very small number of included studies. Since then, many new studies have been performed to examine the effects of H_2_ on endurance, muscle strength, and explosive power (24–26, 33, 34). Therefore, it is urgently demanded to more comprehensively characterize and explicitly examine the effects of H_2_ on physical performance in healthy adults by summarizing the results of the most up-to-date publications.

We have thus conducted a systematic review and meta-analysis based on the available peer-reviewed publications. Only studies with randomized controlled or crossover designs are included, and several subgroup analyses are performed with the goal of providing critical knowledge of the appropriate design of H_2_-based intervention design for the improvement of physical performance.

## 2 Methods

This systematic review and meta-analysis were performed according to the Preferred Reporting Items for Systematic Reviews and Meta-Analysis guideline (35) and registered with PROSPERO (ID CRD42022351559).

### 2.1 Data sources and search strategies

Two authors (K.Z. and Z.S.) independently searched PubMed, Web of Science, Medline, Sport-Discus, Embase, and PsycINFO databases from inception to 10 May 2024. The keywords of the search were as follows: “molecular hydrogen,” “hydrogen rich water,” “hydrogen-rich water,” “hydrogen rich saline,” “hydrogen-rich saline,” “hydrogen gas,” “hydrogen inhalation,” “hydrogen bathing,” “hydrogen-rich calcium powder,” “physical performance,” “athletic performance,” “exercise performance,” “physical exercise,” “aerobic performance,” “aerobic capacity,” “anaerobic performance,” “intermittent exercise,” “sprint,” “strength training,” and “resistance training” (The detailed search strategy is shown in Supplementary Table S1). In addition, a manual search was performed based on the reference lists of selected articles. The search was limited to English only, and no date restrictions were applied.

### 2.2 Selection criteria

To be included in this systematic review, previous studies must meet the following eligibility criteria in accordance with PICOS.

1. Participants: the participants were healthy adults with a mean age of ≥18 years and were free from any dietary supplements or medications while the experiment lasted;
2. Intervention: the intervention was the supplementation of H_2_ by the participants. The source of H_2_ was not limited;
3. Comparator/Control: the control group used placebos that were identical in appearance, texture, and flavor to H_2_ products (e.g., drinking water, air, and capsules);
4. Outcomes: the outcomes include at least one of the measures related to physical performance (e.g., aerobic and anaerobic endurance, muscular strength, lower limb explosive power, subjective fatigue, blood lactate (BLA), and heart rate);
5. Study design: the design of the study was a randomized crossover or randomized controlled trial.

Articles were excluded if they fulfilled the following criteria: 1) animal trials; 2) written in a language other than English or unable to obtain outcome data; 3) review papers and conference articles; and 4) repeated publications.

### 2.3 Data extraction and outcomes

According to the Cochrane Collaboration Handbook, the data extraction process was conducted independently by two authors (C.Y. and Z.S.) (36). The extracted information from the publications included the following: the study (authors and year), sample size, participants (age, height, weight, sex, and training status), methods of H_2_ administration, exercise protocol, and outcome measures. Any outcome measures on which the two authors disagreed were discussed with the other two authors (J.Z. and D.B.) until a consensus was achieved.

The mean and standard deviation of each outcome in post-tests were extracted for each included study. If the post-test values were not available, they were calculated using the following formulas, where the correlation coefficient (Corr) was set at 0.5 (36, 37).


Meanpost=Meanpre+Meanchange

$$\begin{array}{l} \mathrm{SDpost}=\\ \frac{2\times \mathrm{Corr}\times \mathrm{SDpre}+\sqrt{\begin{array}{l} 4\times \mathrm{Corr}2\times \mathrm{SDpre}2-\\ 4\times \left(\mathrm{SDpre}2-\mathrm{SDchange}2\right) \end{array}}}{2} \end{array}$$

If relevant data were missing, we emailed the corresponding author or other authors to request it (36). We extracted relevant data using WebPlotDigitizer (version 4.6) for studies when the data could not be obtained by contacting the authors (38).

Based on the included studies, aerobic endurance, anaerobic endurance, muscular strength, and lower limb explosive power performance were ultimately incorporated into the data synthesis.

The primary outcome of aerobic endurance performance was maximum oxygen uptake (V̇O_2max_) during an incremental load exercise test or peak oxygen uptake (V̇O_2peak_) when V̇O_2max_ was not available (39, 40). The secondary outcome of aerobic endurance performance was aerobic endurance exercise performance, for example, time-to-exhaustion (TTE) or power during incremental load exercise test or fixed-load submaximal test; the time or speed in time trial test (TT).

The primary outcome of anaerobic endurance performance was power output during the 30 s maximal anaerobic test.

The primary outcome of muscle strength was peak torque or force in the maximal voluntary isometric strength test (MVIS) or maximal isokinetic strength test performed pre- or post-high-intensity exercise.

The primary outcome of lower limb explosive power was countermovement jump (CMJ) height, time of short sprint, or peak power output during 10 s maximal effort exercises.

The exploratory outcomes were the rating of perceived exertion (RPE), BLA, and average heart rate (HR_avg_) during physical performance. The RPE, BLA, and HR_avg_ are widely used and are important metrics to characterize subjective fatigue, intensity, and physiologic stress that are closely associated with physical performance (41–43). By exploring the effects of H_2_ on them, it will help more comprehensively characterize the effects of H_2_ supplementation on physical performance.

### 2.4 Quality assessment

Two authors independently evaluated the risk of bias in included studies using the Cochrane Collaboration’s tool (44), which contains six items: 1) selection bias; 2) performance bias; 3) detection bias; 4) attrition bias; 5) reporting bias; and 6) other bias. Each item is categorized into three levels: low-risk bias (green), unclear risk bias (yellow), and high-risk bias (red). Studies were defined as having a high-risk bias if ≥1 item had a high-risk bias. The risk of bias is low if all items are assessed as low risk of bias. Others were assessed as moderate risk of bias. Additionally, the quality of evidence for outcomes was evaluated using the Grading of Recommendations Assessment, Development, and Evaluation (GRADE) (45, 46). The quality of the GRADE evidence was graded as high, moderate, low, and very low based on the quality of study design, quality of implementation, uncertainty of results, and consistency of results (45).

### 2.5 Statistical analysis

Standardized mean difference (SMD) with 95% confidence interval (CI) was used to assess the effect size (ES). ES was classified as trivial (< 0.2), small (0.2 ~ 0.49), moderate (0.5 ~ 0.79), or large (> 0.8) (47). Meta-analysis was performed in Stata v15.1 (STATA Corp., College Station, TX) using the inverse-variance method. The *I*^2^ statistic was used to evaluate heterogeneity among the trials with the following criteria: trivial (< 25%), low (25 ~ 50%), moderate (50 ~ 75%), and high (> 75%) (48). A random-effects model was used to estimate pooled effects, as heterogeneity was anticipated across studies due to differences in participants and interventions. Subgroup analysis was used to explore potential sources of heterogeneity (49). The Funnel plots and Egger tests were used to evaluate publication bias. If potential publication bias was detected, we used the trim and fill method for the sensitivity analysis of the results (50). All the statistical significance was set at a *p*-value of <0.05.

## 3 Results

### 3.1 Study selection

**Figure 1:**
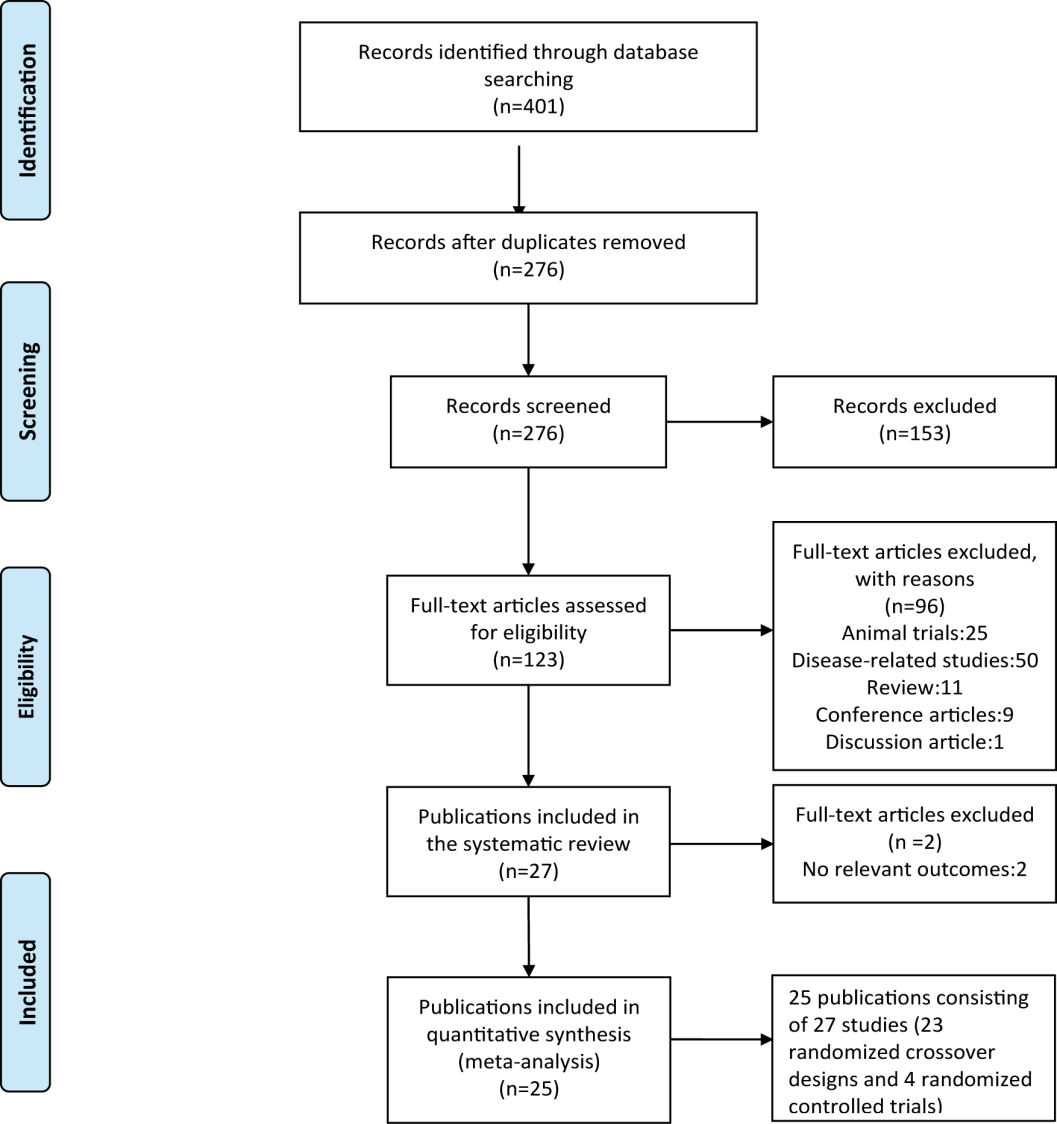
Study flowchart.

The screening procedure of the included studies is shown in Figure 1. A total of 401 potentially relevant publications were retrieved (PubMed *n* = 77, SPORT-Discus *n* = 65, Medline *n* = 71, Web of Science *n* = 89, PsycINFO *n* = 5, and Embase = 94). Based on the criteria above, 248 publications were discharged after reviewing the titles and abstracts. After evaluating the full texts, 27 publications (29 studies) were included in the systematic review. Finally, 25 publications consisting of 27 studies (23 randomized crossover designs and 4 randomized controlled trials) were included in the quantitative synthesis (Table 1). One study (28) included two randomized controlled trials, and the other study (23) included a randomized crossover trial and a randomized controlled trial.

**Table 1:** Characteristics of the included studies (*n* = 29).

Study	Design	Sample size	Age (yr)	%F	Training status	Methods of H_2_ administration	Exercise protocol	Outcome measures
Aoki et al. (51)	RCD	10	20.9 ± 1.3	0	Elite soccer players	HRW (H_2_ conc.:0.92 ~ 1.02 ppm) Three 500 mL doses before exercise	Cycling for 30 min at 75% V̇O_2max_ and maximal isokinetic knee extensions test	Peak torque→; BLA↓; d-ROMs→; BAP→; CK→; MF→; MPF→
Ostojic et al. (52)	RCT	H:26	25.1 ± 3.4	0	Male athletes	HRW (pH:9.3; ORP: −372 mV; EC:12.0 ms/m; DO:6.0 mg/L) 2 L per day for 2 weeks	15 min incremental treadmill running (start at 8 km/h and increase by 2 km/h every 3 min).	Blood pH→; Partial pressure for carbon dioxide→; Serum bicarbonates↑
		P:26	23.8 ± 4.5					
Drid et al. (53)	RCD	8	21.4 ± 2.2	100	Judo athletes	HRW^﹟^ 300 mL within 30 min before exercise	Special judo fitness test	Fitness performance index→; BLA↓; Blood pH→; Bicarbonate↓; HR_max_→; HR_recovery_→
Kawamura et al. (54)	RCD	9	25.0 ± 3.0	0	Healthy and active young men	HRW bathing for 20 min before exercise	Downhill running (8% decline at 75%VO_2_peak for 30 min)	Running speed→; VO_2_→; %VO_2peak_→, HR_avg_→; BLA→; VAS→; CK→; Myoglobin; Malondialdehyde→; d-ROMs→; BAP↓; Myeloperoxidase→; Interleukin-6→
Da Ponte et al. (55)	RCD	8	41 ± 7	0	Well-trained cyclists	HRW^﹟^ (pH:9.8; ORP: −180 mV; FH:450 ppb; TDS:180 mg/L) 2 L per day for 2 weeks before exercise	30 min intermittent(10x3min) cycling	Pm for 30min^a^→; BLA→; Fatigue index→; RPE→; V̇O_2_→; RER→; HR_avg_→; Blood pH→; Bicarbonate [HCO_3_-] →; Base excess→; pO_2_→; pCO_2_→; Hemoglobin→; Hemoglobin Sat→; Glucose→
LeBaron et al. (22)	RCD	19	25.0 ± 8.9	21	Untrained healthy participants	HRW^﹟^ (TDS:13.1 mg/L) 500 mL intake the day before and on the day of exercise	Incremental treadmill running test to exhaustion	V̇O_2peak_→; HR_avg_↓; RER→; RR→
Botek et al. (56)	RCD	12	27.1 ± 4.9	0	Recreationally trained sports science students	HRW (pH:7.4; ORP: -400 mV; Temp: 22°C; H_2_ conc.:0.5 ppm)	Incremental cycling test to exhaustion	BLA↓; RPE↓; V̇E→; V̇O2→; V̇E/V̇O_2_↑; HR_max_→; RQ→
						600 mL within 30 min before exercise		
Javorac et al. (57)	RCD	20	22.9 ± 1.5	50	Untrained physically active participants	HRG^﹟^ (%4 H_2_) 20 min once-per-day inhalation for 7 days	Maximal voluntary isometric strength of leg, YMCA bench press test, and incremental treadmill running test to exhaustion	TTE→; Maximum running speed↑; V̇O_2max_→; MVIS→; YMCA endurance→; Resting BLA→; Blood pressure→; Resting HR→; MRS↑; Insulin→; Ghrelin→; IGF-1↑; CK→; Myoglobin→; C-reactive protein↑; Ferritin↑; ESR→
Ooi et al. (29)	RCD	14	34 ± 4	0	Well-trained runners/triathletes	HRW (H_2_ conc.: 2.60 ppm) 2 doses of 290 mL within 5 ~ 10 min before exercise	Incremental treadmill running test to exhaustion	TTE→; Speed at OBLA→; V̇O_2max_→; BLA→; RPE→; HR_max_→; RE→; V̇E_max_→; RER→; Blood Glucose→; Blood HCO3-→; Blood pH→
Mikami et al. (Exp.1) (28)	RCT	H:52	51.2 ± 6.9	55.8	Untrained physically active participants	HRW (H_2_ conc.:0.8 ppm) 500 mL within 30 min before exercise	Incremental cycling test to 75% HRmax	V̇O_2max_→; Resting RPE↓; VAS↓; HR_avg_^a^↓
		P:47	51.5 ± 7.9	57.4				
Mikami et al. (Exp.2) (28)	RCT	H:30	43.6 ± 13.3	50	Fitness trainers	HRW (H_2_ conc.:1.0 ppm) 500 mL within 10 min before exercise	Incremental cycling test to HRmax	V̇O_2max_↑; RPE↓
		P:30	43.2 ± 14.4	50				
Dobashi et al. (30)	RCD	8	19.4 ± 0.85	0	Untrained physically active participants	HRW (Temp: 4°C; H_2_ conc.:5.14 ppm) 500 mL within 5 min before and after the exercise for 3 days	Three sets of 10-s repeated sprint cycling over 6 min, CMJ, and MVIS of knee extensions test	BLA→; MVIS→; CMJ→; Pmax for 10 s → ; Pm for 10 s→; d-ROMs→; BAP→
Botek et al. (58)	RCD	16	31.6 ± 8.6	0	Well-trained runners	HRW (pH:7.8; H_2_ conc.: 0.9 ppm) 420 mL doses at 24 h, 3 h, 2 h, and 40 min before exercise	4.2-km up-hill race	Race time→; RPE→; HR_avg_→
Shibayama et al. (27)	RCD	8	20.9 ± 0.3	0	Untrained physically active participants	HRG^﹟^ (68% H_2_) 60 min after exercise	30 min treadmill running (75%V̇O_2max_), CMJ, 10 s sprint cycling, 30s sprint cycling, and MVIS of knee extensions test	Pm for 30s→; MVIS→; CMJ↑; Pmax for 10s→; d-ROMs→; BAP→; U8ER↓; CKa→; LDa→; White blood cells→
Todorovic et al. (59)	RCD	6	24 ± 4	0	Healthy and active young men	HRW bathing (dissolve magnesium malate effervescent tablets in 200 L of tap water in a bathtub) immerse the whole body for 30 min immediately after exercise.	Five sets x 10 reps of eccentric leg presses (120% 1RM) followed by two sets x 10 reps of eccentric leg presses (100% 1RM), with 3 min between sets.	VAS↓; CK↓; Lactate dehydrogenase→; Aldolase→; Aspartate transaminase→; Troponin I→; Myoglobin→; White blood cells→; C-reactive protein→
Hori et al. (60)	RCD	12	21.8 ± 5.8	0	Untrained healthy participants	HRG (1% H_2_) inhalation of H_2_ gas 10 min before and 20 min during exercise	Cycling for 30 min at 60% V̇O_2peak_	VCO_2_↑; VE↑; HRavg ^a^→; Resting HR → Vacetone↑; V̇O_2_ rest→; VCO_2_ rest→; VE rest→; Vacetone rest→; d-ROMs→; BAP→
Hori et al. (Exp.1) (23)	RCD	9	19.9 ± 1.2	33.3	Untrained university students	HRW (H_2_ conc.:4.3 ppm) 500 mL doses at 35 min before exercise	Incremental cycling test to exhaustion	V̇O_2peak_↑; Peak load→; BLA→; RPE→; HRmax→; Resting HR→; CDO→; RER→; V̇E→; d-ROMs→; BAP→
Hori et al. (Exp.2) (23)	RCT	H:10	20.3 ± 1.3	0	Untrained university students	HRW (H_2_ conc.:5.9 ppm) 500 mL on all weekdays for 2 weeks	Incremental cycling test to exhaustion	V̇O_2peak_→; BLA ^a^→; Peak load→; RPE→; CDO→; RER→; V̇E→; HR_max_→; Resting HR→; d-ROMs↑; BAP↑
		P:10	20.4 ± 4.7					
Botek et al. (34)	RCD	12	23.8 ± 1.9	0	Resistance trainers	HRW (Temp: 22°C; pH:7.8; ORP: −652 mV; H_2_ conc.: 0.9 ppm) 210 mL at 30 min and at 1 min before training, 210 mL in the middle of the exercise session, then another 210 mL immediately after the end of the exercise session, and 420 mL of HRW at 30 min of recovery	A half squat, knee flexion, and extension exercises with the load set at 70%1RM for 3 sets (10 reps/set) + Lunges were performed with a load of 30% of body mass for 3 sets (20 reps/set)	RPE→; BLA↓; VAS↓; CK→; CMJ→; HRV→; Time of lunges↑
Timon et al. (26)	RCD	27	25.9 ± 5.6	Un	Recreationally trained cyclists (n = 12) and untrained participants (n = 15)	HRW (pH: 7.5; H_2_ conc.:1.9 ppm; ORP: -600 mV) 1920 and 2,240 mL per day for 7 days	Incremental cycling test to exhaustion and 30 s maximal anaerobic test	V̇O_2max_↑; TTE↑; Pm of maximal anaerobic test↑; BLA→; Pmax of maximal cycling test↑; Fatigue index↓; RPE→; HR_max_→; VT2%V̇O_2max_↑
Alharbi et al. (31)	RCD	18	21 ± 1	0	Recreationally trained participants	HRC^﹟^ (0.636 μg/capsule) 2.544 μg/day for 3 days	Incremental cycling test to exhaustion	V̇O_2peak_→; TTE→; BLA→; P_max_→; HR_max_→; Electrolytes (Na + →; K + →; Ca2 + →; Cl − →; AGap↑; AGapK→); V̇E↑; V̇O_2_↑; V̇CO_2_↑; Blood gas (pH↑; PO_2_→; PCO_2_→; HCO_3_-↑); TR-NIRS in the RF/VL (Total [Hb + Mb] →; Deoxy [Hb + Mb] ↑; StO_2_↑)
Dong et al. (24)	RCT	H:9	23.22 ± 1.09	33	Dragon boat athletes	HRW^﹟^ (FH:1600 ppb) 1,000 mL per day for 8 days	30 s maximal dynamometer rowing test	Predicted time of rowing 500 m→; Pm→; Pmax↑; HRmax↓; HR_recovery_↓; Resting HR→;
		P:9	22.67 ± 0.87					
Botek et al. (25)	RCD	16	18.8 ± 1.2	0	Professional soccer players	HRW (pH:7.9 ORP: −652 mV; Temp: 20°C; H_2_ conc.:0.9 ppm) 420 mL at 120 min, 60 min and 210 mL at 15 min, and 5 min before exercise	Repeated sprints (15 × 30 m track sprints with recovery 20 s)	15th 30-meter sprint time↓; BLA → RPE→
Valenta et al. (33)	RCD	24	17.5 ± 1.8	0	Trained track and field runners	HRW (pH:7.8; ORP: -600 mV; H2 conc.:0.9 ppm) 420 mL was applied 120 min and 60 min before exercise, and 210 mL was applied 30 min and 10 min before exercise	Individual maximal aerobic speed until exhaustion (the time to exhaustion)	TTE→; DTE→; BLA→; HR_max_→; BF→; VE→; VO2→; VCO2→; VE/VO2→; RQ→
Alharbi et al. (61)	RCD	10	20.0 ± 1.0	0	Trained track and field runners	HRC (0.636 μg/capsule) 2.544 μg supplements 1 h before exercise	Repeated cycling (Six repetitions of the 7 s all-out pedaling at 7.5% body weight separated by 40 s intervals)	Pmax↑; Muscle deoxygenation↑; Tissue O_2_ saturation↑; HR_max_^a^→; HR_recovery_^a^→; Blood pH↑
Hong et al. (62)	RCD	24	21.3 ± 2.7	0	Physical education students	HRG (The ratio of oxygen to hydrogen in the H_2_ gas is 2:1); Inhaled H_2_ gas for 20 min before exercise	Constant workload cycling exercise; MVIS of knee extensions test	RPE↓; HR↓; PFC↑; MVIS→
Jebabli et al. (63)	RCD	22	21 ± 1	0	Amateur middle-distance runners	HRW (Temp: 12°C; pH:7.4; H_2_ conc.: 0.55–0.65 mmol) 500 mL before exercise	Vameval test and race with maximal aerobic speed until voluntary exhaustion	Speed of the Vameval test↑; TTE↑; SJ→, CMJ→; 5JT→; RPE↑; HR_max_↑
Dong et al. (64)	RCD	24	21.3 ± 2.7	0	Healthy adult men	HRG (The ratio of oxygen to hydrogen in the H_2_ gas is 2:1); Inhaled H_2_ gas for 60 min before exercise	Ride T_max_ at 80% W_max_ on cycle ergometers	RPE↓^a^; VAS↓; CMJ→; BLA↓; OH-↑; GSH-PX→
Sládečková et al. (65)	RCD	12	f:21.5 ± 5.0 m:18.9 ± 1.3	66.6	Elite swimmers	HRW (Temp: 20/20°C; pH 7.9/7.7; OPR: −652/+170 mV) 2,520 mL (1,260 mL/day) 3 days before the sessions and 2,520 mL on the experimental day	Morning session:4 × 50 m x 3 sets; Afternoon session:400 m dash	VAS↓; CK↓; CMJ↑

a Outcome data were not available by contacting the corresponding author and other authors on the publication; AGap, anion gap; AGapK, anion gap potassium; BLA, blood lactate; BF, breathing frequency; BAP, biological antioxidant potential; CK, creatine kinase; CKa, creatine kinase activity, CMJ, countermovement jump; CDO, carbon dioxide output; d-ROMs, diacron-reactive oxygen metabolites; DO, dissolved oxygen; DTE, distance to exhaustion; Exp., experiment; EC, electric conductivity; ESR, erythrocyte sedimentation rate; f, female; FH, free hydrogen; GSH-PX, glutathione peroxidase activity; H, H_2_; H_2_ conc., H_2_ concentration; HRW, hydrogen-rich water; HRG, hydrogen-rich gas; HRC, hydrogen-rich calcium powder; HRG: hydrogen-rich gas; HR, heart rate; HRV, HRmax, maximal heart rate; HRavg, average heart rate; HR recovery, recovery heart rate; heart rate variability; LDa, lactate dehydrogenase activity; m, male; MF, median frequency; MPF, mean power frequency; MVIS, maximal voluntary isometric strength; MRS, maximal running speed; ORP, oxidation reduction potential; OḤ-, the ability to inhibit hydroxyl radicals; OBLA, onset of blood lactate accumulation at 4 mmol∙L-1; P, placebo; Pm, mean power; Pmax, maximum power; PFC, prefrontal cortex activation; RCD, randomized crossover design; RCT, randomized controlled trial; RE, running economy; rep., repetitions; RER, respiratory exchange ratio; RR, respiratory rate; RQ, respiratory quotient; RPE, rating of perceived exertion; RF, rectus femoris muscle; SCKa, serum creatine kinas activity; SLDa, serum lactate dehydrogenase activity; Temp, temperature; TTE, time-to-exhaustion; TDSs, total dissolved solids; TR-NIRS, time-resolved near-infrared spectroscopy; T_max_, maximum cycling time; U8ER, urinary 8-hydroxydeoxyguanosine excretion rate; Un, unreported; V̇O_2max_, maximum oxygen uptake; V̇O_2peak_, peak oxygen uptake; V̇O_2_, oxygen uptake; VT2 %V̇O_2max_, percentage of maximal oxygen uptake in the ventilatory anaerobic threshold; V̇E, ventilation volume; VASs, visual analogue scales; VL, vastus lateralis muscle; W_max_, maximum cycling power; %F, %female; 5JT, five jump test; f, female; m, male; SJ, squat jump test; ↓, H_2_ significantly (*p* < 0.05) reduced the outcome compared to placebo; ↑, H_2_ significantly (*p* < 0.05) improved the outcome compared to placebo; →, no significant difference (*p* > 0.05) between H_2_ and placebo; ^﹟^, unreported molecular hydrogen concentration.

### 3.2 Characteristics of included studies

#### 3.2.1 Participant characteristics

A total of 597 participants, with mean ages ranging from 17.5 to 51.5 years, were included. The training status of these participants was classified according to the included studies as untrained (*n* = 224) and trained (*n* = 373), with 215 of them being well-trained athletes (e.g., professional soccer players and elite runners).

#### 3.2.2 Methods of H_2_ administration

A gold standard regimen for H_2_ application does not appear to exist. The included studies implemented four sources of H_2_, that is, drinking HRW (*n* = 18) (22–26, 28–30, 33, 34, 51–53, 55, 56, 58, 63, 65), HRW bathing (*n* = 2) (54, 59), inhalation of H_2_-rich gas (HRG) (*n* = 5) (27, 57, 60, 62, 64), and oral ingestion of H_2_-rich calcium (HRC) powder (*n* = 2) (31, 61). H_2_ concentrations were found to be highly variable (e.g., HRW:0.5 ~ 5.9 ppm; HRG:1 to 68%) among the various products examined. Nine studies did not report the concentration of H_2_ (22, 24, 27, 31, 53–55, 57, 59). Single (*n* = 9) or multiple doses (ranging from 3 to 4 doses) of H_2_ supplementation prior to exercise is a common intervention protocol. In total, 14 studies examined the effects of H_2_ intake within 24 h before exercise (23, 25, 28, 29, 33, 51, 53, 54, 56, 58, 61–64). Nine studies implemented the protocol of repeated intake of H_2_ from 2 to 14 days before exercise (22–24, 26, 30, 31, 52, 55, 57). One study (60) used 30 min inhalation of HRG during exercise. Another study (65) examined the effects of multiple doses of H_2_ supplementation before and during exercise. Two studies examined the effects of a single intake of H_2_ after exercise (27, 59). One study (34) used 210 mL at 30 min and 1 min before exercise, 210 mL during mid-exercise, another 210 mL immediately after exercise, and 420 mL of HRW 30 min after recovery. The physicochemical properties of HRW, HRW bathing, HRG, and HRC are shown in Table 1. Placebos were identical in appearance, texture, and taste to H_2_ products, such as drinking water, air, and capsules.

#### 3.2.3 Exercise protocol and outcome measurements

The included studies highlighted the effects of H_2_ supplementation on aerobic endurance, anaerobic endurance, muscular strength, and lower extremity explosive strength in participants. In these studies, continuous incremental load and fixed-load subliminal exercise were the most commonly used aerobic endurance intervention or testing protocols. V̇O_2max_, V̇O_2peak_, TTE, race time, and power were metrics used to measure aerobic endurance performance (24, 26, 28, 29, 33, 55, 57, 61, 63). The 30 s maximal anaerobic power test (i.e., pedaling bicycle or rowing dynamometer) was used to assess the anaerobic endurance (i.e., mean or maximal power) (24, 26, 27). One study (57) used the MVIS to assess the force of knee extension prior to high-intensity aerobic exercise; four studies (27, 30, 51, 62) were conducted to evaluate the magnitude of knee extensor force or peak torque in the MVIC after vigorous exercise. Eight studies (25, 27, 30, 34, 61, 63–65) evaluated alterations in lower limb explosive power (i.e., CMJ height and peak power output during 10 s or 30 m sprint) during or after vigorous exercise in participants. One study (53) used the special fitness test to assess the effects of HRW intake on athletic performance in judo athletes. Additionally, the included studies focused on assessing the effects of H_2_ administration on various physiological parameters during exercise, such as RPE, BLA, HR, pH, respiratory function, antioxidant levels, muscle oxygenation, and endocrine system. The outcomes of each study are summarized in Table 1.

### 3.3 Quality assessment

**Figure 2:**
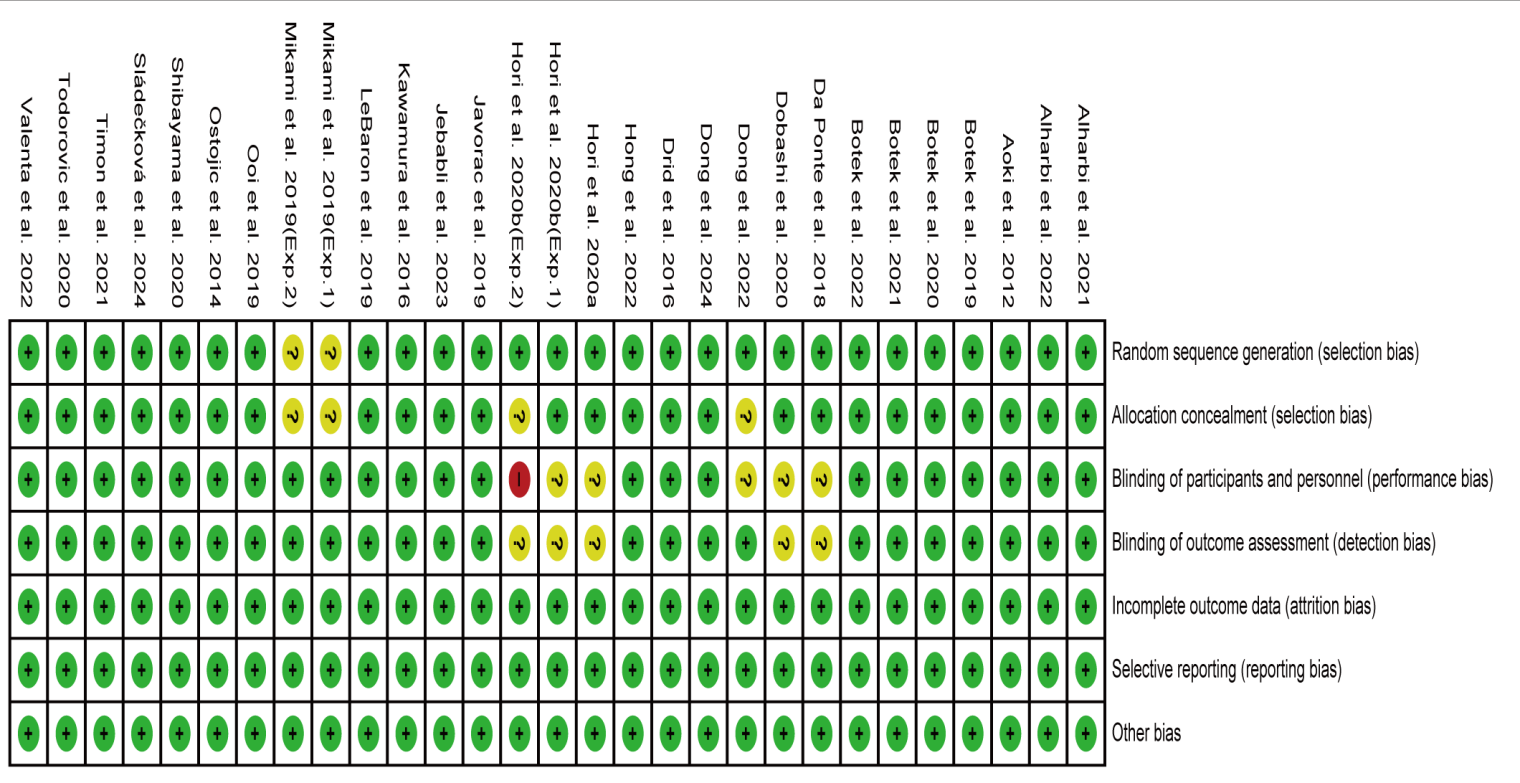
Risk of bias in the included studies.

The risk of bias in the 27 publications (29 studies) was assessed, and a consensus was reached after discussion. The overall result is shown in Figure 2. Two studies (23, 28) did not adequately report on participant randomization and concealment methods. Five studies (23, 24, 30, 55, 60) did not adequately describe participant, staff, or evaluator blinding. No studies had incomplete results due to participants’ withdrawal. All studies reported experimental procedures and conducted the experiments as planned. According to the possibility of bias, the study was assessed as being low risk, moderate risk, or high risk. One study (23) was evaluated as having a high-risk bias, five studies (24, 28, 30, 55, 60) had a moderate risk bias, and others were assessed as having a low-risk bias. The quality of evidence for outcomes was evaluated as moderate to high, and details for the evaluation of the GRADE framework are presented in Supplementary Table S2.

### 3.4 Meta-analysis

A subgroup analysis was performed on aerobic endurance, anaerobic endurance, muscle strength, lower limb explosive power, RPE, and BLA, considering potential sources of heterogeneity, including exercise types and H_2_ sources. Additionally, we used a subgroup analysis to explore the effects of H_2_ supplementation on muscle performance before or after vigorous exercise (Table 2).

**Table 2:** Subgroup analysis results regarding the effects of H_2_ on RPE and BLA.

Outcomes	Variables	No. of studies	SMD (95% CI)	*p-*value	Test of heterogeneity		
					*χ^2^*	*p*-value	*I^2^* (%)
RPE	Exercise types						
	Strength training	1	−1.41 (−2.32, −0.50)	0.002	0	—	—
	Repeated sprints	1	−0.96 (−1.70, −0.22)	0.011	0	—	—
	Aerobic endurance exercise	10	−0.33 (−0.59, −0.07)	0.013	15.29	0.083	41.2
	Anaerobic endurance exercise	1	0.29 (−0.25, 0.83)	0.290	0	—	—
	Hydrogen source						
	HRW	12	−0.32 (−0.60, −0.03)	0.029	25.13	0.009	56.2
	HRG	1	−0.91 (−1.51, −0.31)	0.003	0	—	—
BLA	Exercise types						
	Strength training	1	−0.53 (−1.34, 0.29)	0.206	0	—	—
	Repeated sprints	2	−0.20 (−0.77, 0.37)	0.496	0.99	0.320	0
	Aerobic endurance exercise	9	−0.38 (−0.67, −0.08)	0.013	12.08	0.148	33.8
	Anaerobic endurance exercise	2	−0.67 (−1.72, 0.38)	0.213	3.01	0.083	66.8
	Hydrogen source						
	HRW	12	−0.37 (−0.59, −0.16)	0.001	15.37	0.166	28.4
	HRG	1	−0.47 (−1.04, 0.11)	0.111	0	—	—
	HRC	1	0.00 (−0.65, 0.65)	0.999	0	—	—

RPE, Rating of perceived exertion; BLA, blood lactate; HRW, hydrogen-rich water; HRG, hydrogen-rich gas; HRC, hydrogen-rich calcium powder.

#### 3.4.1 Effects of H_2_ on aerobic endurance

##### 3.4.1.1 V̇O_2max_ (V̇O_2peak_)

Three studies (23, 26, 28) showed that H_2_ can significantly improve V̇O_2max_ or V̇O_2peak_ as compared to the placebo; while another five publications (six studies) (22, 28, 29, 31, 57) showed opposite results: H_2_ cannot significantly improve V̇O_2max_ or V̇O_2peak_ (Table 1).

**Figure 3:**
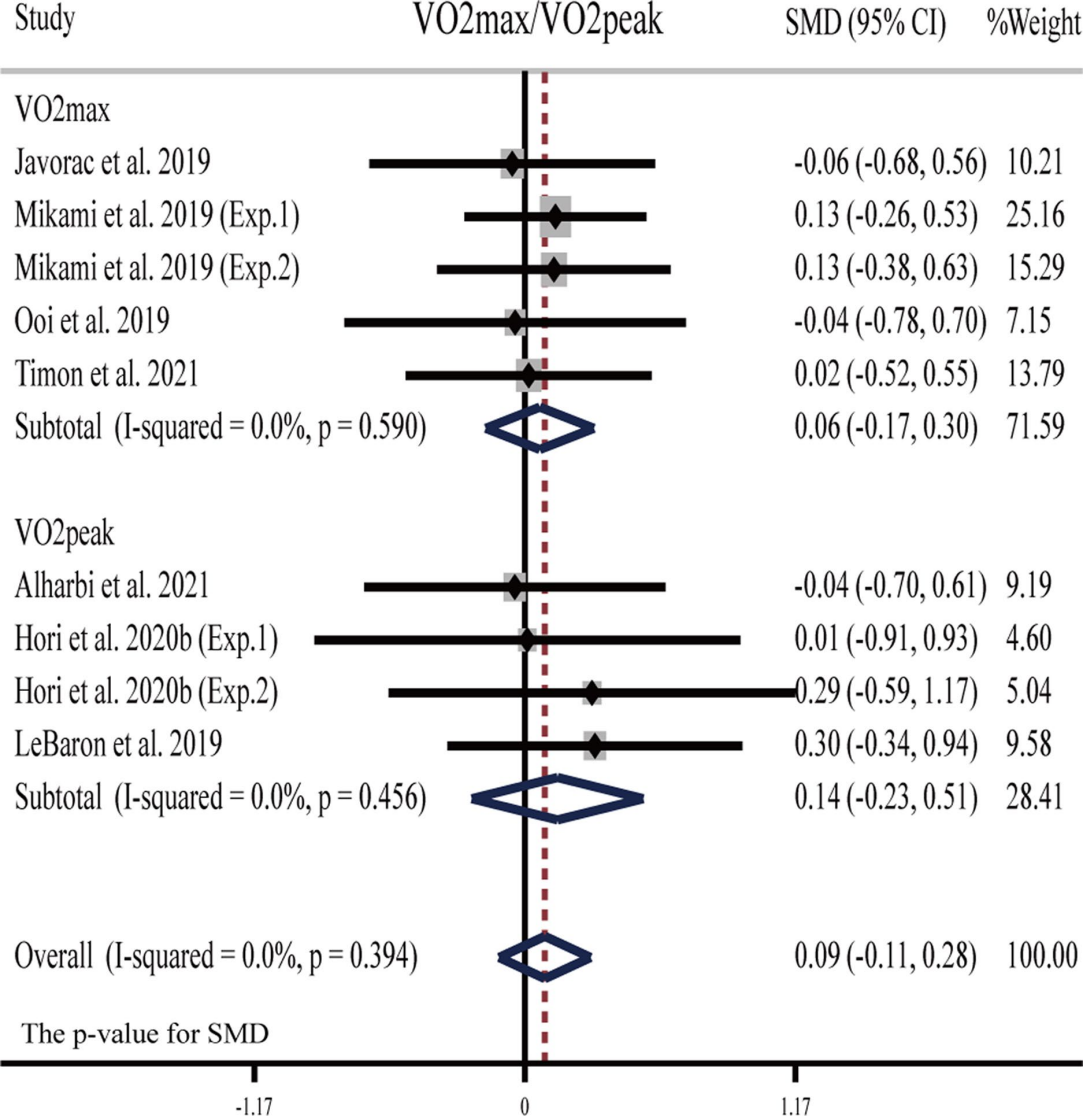
Forest plot of the effects of H_2_ supplementation on V̇O_2max_/V̇O_2peak_. Exp.1, Experiment 1; Exp.2, Experiment 2.

The pooled ES of V̇O_2max_ and V̇O_2peak_ was not significant and trivial (SMD = 0.09, 95% CI −0.11 to 0.28, *p* = 0.394, Figure 3) and without heterogeneity (*I*^2^ = 0%, *p* = 0.996). The funnel plot (Supplementary Figure S1A) and Egger’s test (t = −0.30, *p* = 0.776) indicated that there was no publication bias. Subgroup analyses showed non-significant trivial ESs for HRG (SMD = -0.06, 95% CI −0.68 to 0.56, *p* = 0.861), HRC (SMD = −0.04, 95% CI −0.70 to 0.61, *p* = 0.895), and HRW (SMD = 0.12, 95% CI −0.10 to 0.34, *p* = 0.290) on V̇O_2max_ (V̇O_2peak_).

##### 3.4.1.2 Aerobic endurance exercise performance

Two studies (26, 63) showed that H_2_ can significantly improve aerobic exercise performance as compared to the placebo, while another eight publications (nine studies) (23, 24, 29, 31, 33, 54, 57, 58) showed that H_2_ cannot (Table 1).

**Figure 4:**
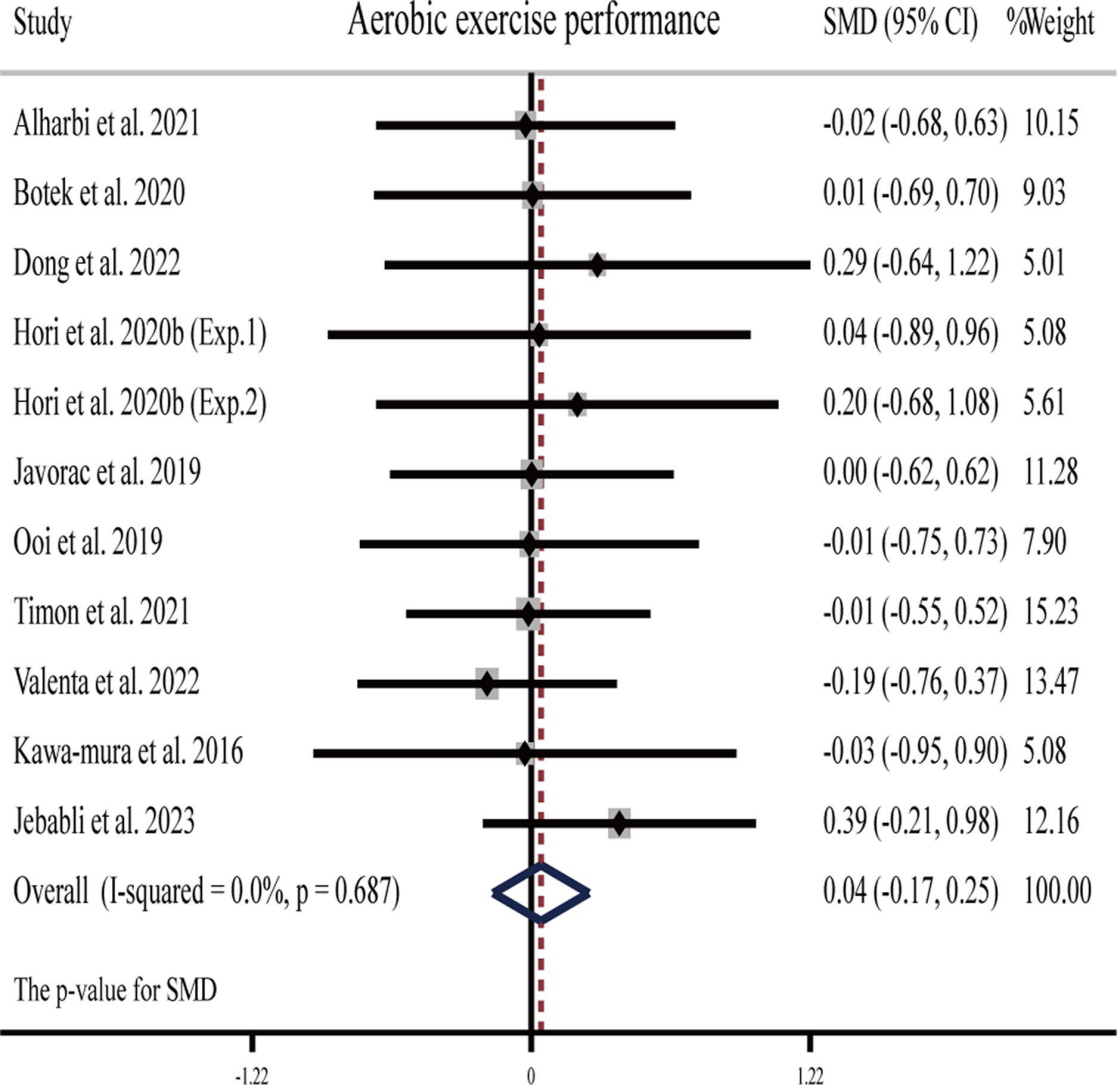
Forest plot of the effects of H_2_ supplementation on aerobic exercise performance. Exp.1, Experiment 1; Exp.2, Experiment 2.

The pooled ES of aerobic exercise performance was not significant and trivial (SMD = 0.04, 95% CI -0.17 to 0.25, *p* = 0.687, Figure 4) and without heterogeneity (*I*^2^ = 0%, *p* = 0.991). The funnel plot (Supplementary Figure S1B) and Egger’s test (*t* = 0.75, *p* = 0.474) indicated that there was no publication bias on these results. Subgroup analyses showed non-significant trivial ESs for HRG (SMD = 0.002, 95% CI −0.618 to −0.622, *p* = 0.994), HRC (SMD = −0.02, 95% CI −0.68 to 0.63, *p* = 0.941), and HRW (SMD = 0.06, 95% CI −0.18 to 0.29, *p* = 0.632) on aerobic exercise performance.

#### 3.4.2 Effects of H_2_ on anaerobic endurance

One study (26) showed that H_2_ can significantly improve mean and peak power output during the 30 s maximal anaerobic test as compared to the placebo. One study (24) showed that H_2_ can significantly improve peak power output in a 30 s maximal anaerobic test compared to placebo but cannot significantly improve mean power, while another study (27) showed the opposite result that H_2_ cannot significantly improve mean power output during the 30 s maximal anaerobic test (Table 1).

**Figure 5:**
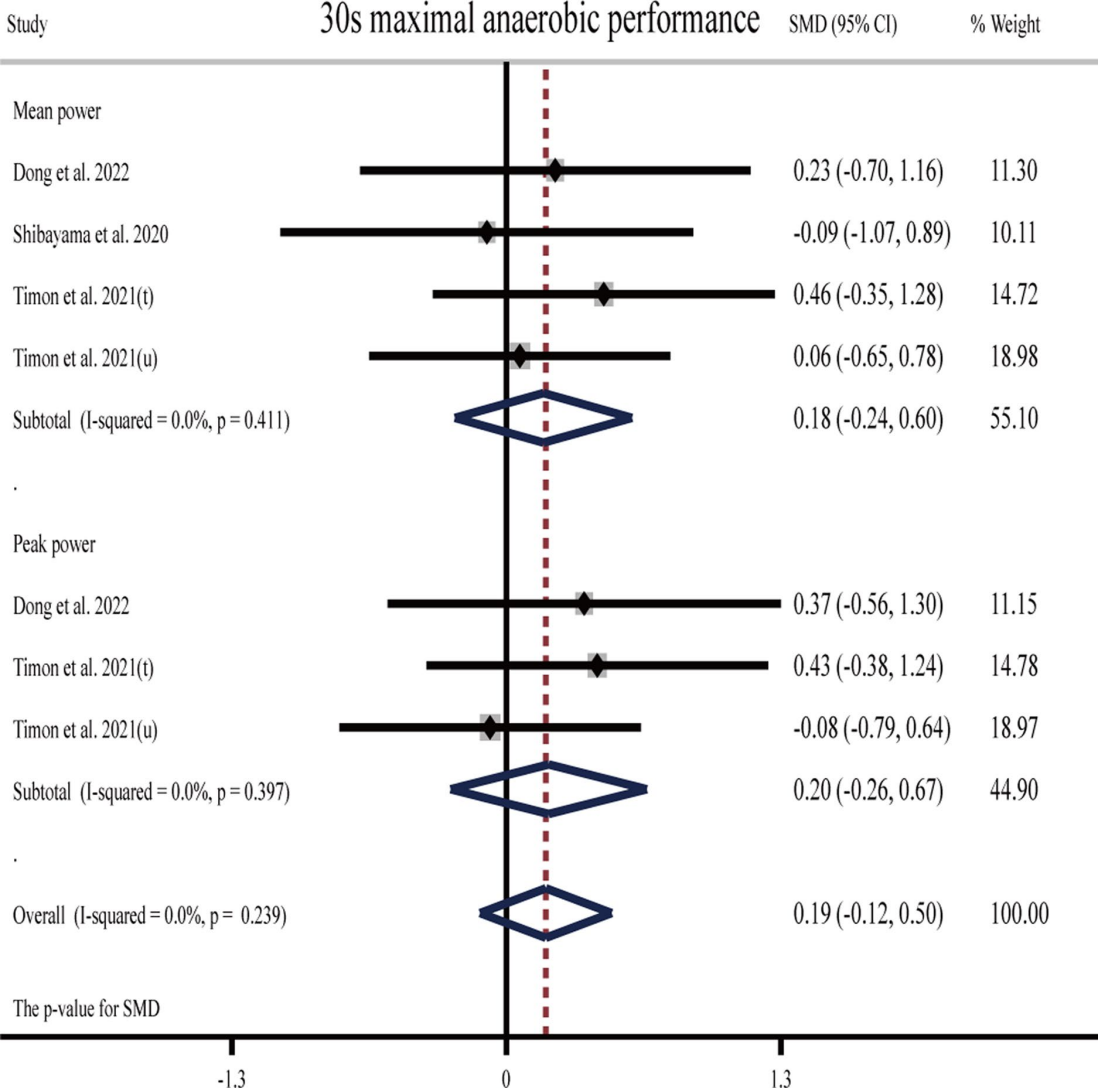
Forest plot of the effects of H_2_ supplementation on anaerobic exercise performance. t, trained participants; u, untrained participants.

The pooled ES of anaerobic exercise performance was not significant and close to small (SMD = 0.19, 95% CI −0.12 to 0.50, *p* = 0.239, Figure 5) with low heterogeneity (*I*^2^ = 0%, *p* = 0.929). The funnel plot (Supplementary Figure S1C) and Egger’s test (*t* = 0.58, *p* = 0.586) indicated that there was no publication bias. With regard to the source of H_2_, the ES was trivial for HRG (SMD = −0.09, 95% CI −1.07 to 0.89, *p* = 0.853), while it was small (SMD = 0.22, 95% CI −0.11 to 0.55, *p* = 0.192) for HRW.

#### 3.4.3 Effects of H_2_ on muscle strength

Five studies (27, 30, 51, 57, 62) showed that H_2_ cannot significantly improve maximum strength compared to the placebo (Table 1).

**Figure 6:**
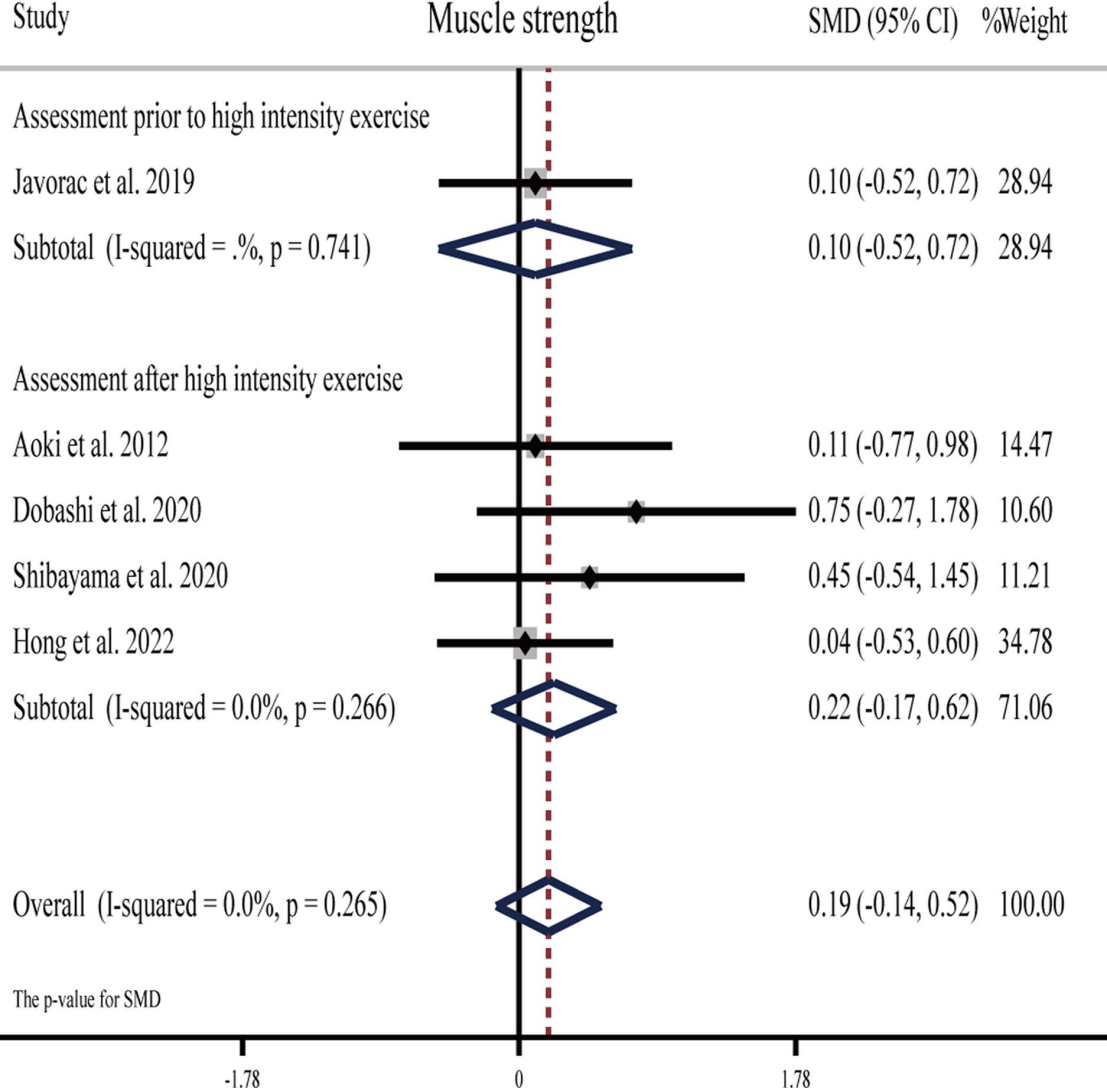
Forest plot of the effects of H_2_ supplementation on muscle strength.

The pooled ES of muscle strength was not significant and close to small (SMD = 0.19, 95% CI -0.14 to 0.52, *p* = 0.265, Figure 6) and with low heterogeneity (*I*^2^ = 0%, *p* = 0.770). The funnel plot (Supplementary Figure S1D) and Egger’s test (*t* = 2.67, *p* = 0.076) indicated no publication bias.

Subgroup analyses showed that the ES was trivial (SMD = 0.10, 95% CI −0.52 to 0.72, *p* = 0.741) for muscle strength assessed before vigorous exercise, and it was small (SMD = 0.22, 95% CI −0.17 to 0.62, *p* = 0.266) for H_2_ on muscle strength assessed after vigorous exercise. With regards to the source of H_2_, the ES was not significantly trivial for HRG (SMD = 0.13, 95% CI −0.26 to 0.51, *p* = 0.520), while it was small (SMD = 0.38, 95% CI −0.29 to 1.05, *p* = 0.265) for HRW.

#### 3.4.4 Effects of H_2_ on lower limb explosive power

Three studies (25, 61, 65) showed that H_2_ can significantly improve lower limb explosive power as compared to the placebo, while another five studies (27, 30, 34, 63, 64) showed opposite results that H_2_ cannot improve lower limb explosive power (Table 1).

**Figure 7:**
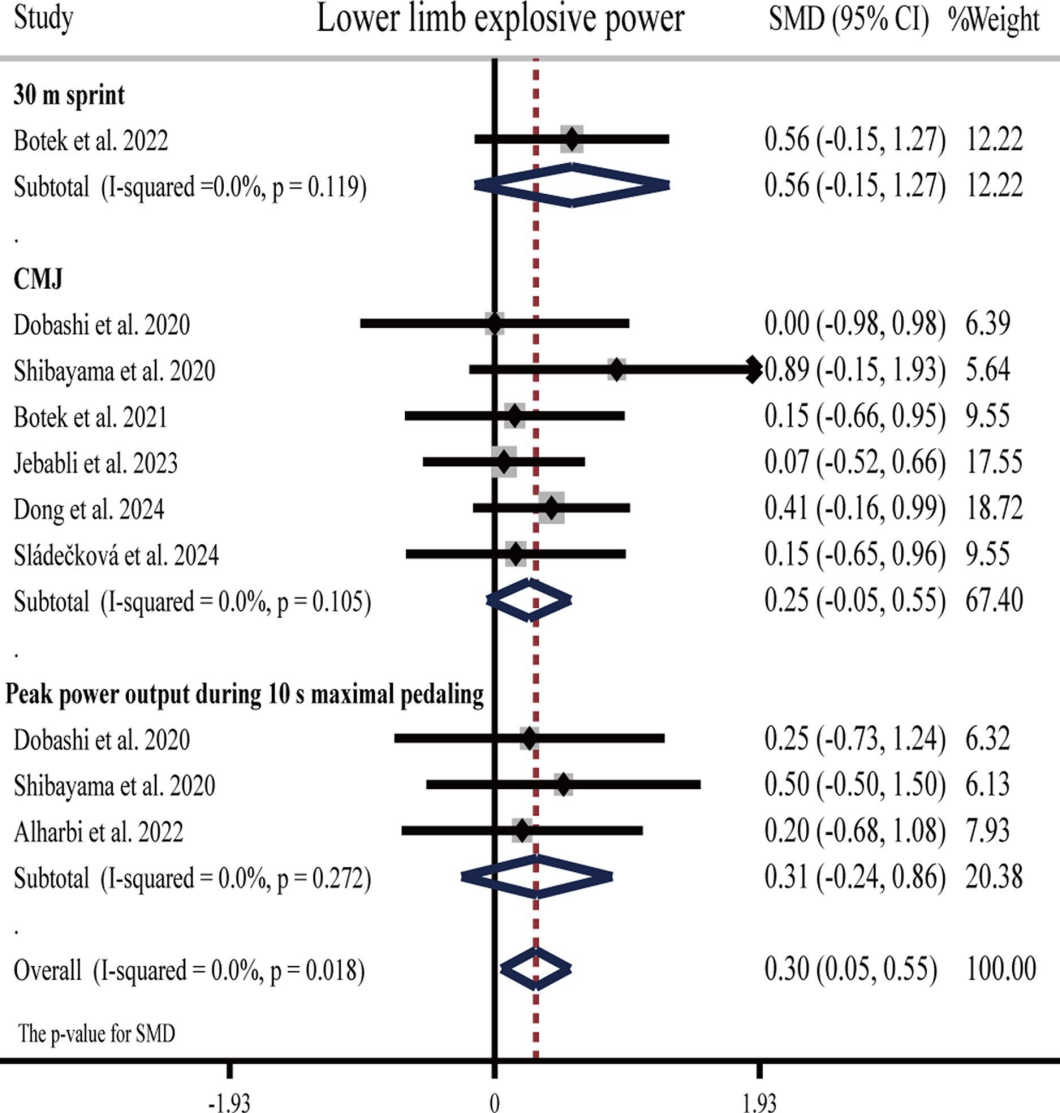
Forest plot of the effects of H_2_ supplementation on lower limb explosive power.

The pooled ES of lower limb explosive power was significant and small (SMD = 0.30, 95% CI 0.05 to 0.55, *p* = 0.018, Figure 7) and without heterogeneity (*I*^2^ = 0%, *p* = 0.949). The funnel plot (Supplementary Figure S1E) and Egger’s test (*t* = 0.49, *p* = 0.636) indicated no publication bias. Subgroup analyses showed that the ES of HRG on lower limb explosive power was significant and moderate (SMD = 0.52, 95% CI 0.07 to 0.97, *p* = 0.023), while HRC was not significant and small (SMD = 0.20, 95% CI −0.68 to 1.08, *p* = 0.655), and HRW was not significant and small (SMD = 0.20, 95% CI −0.11 to 0.52, *p* = 0.206).

#### 3.4.5 Effects of H_2_ on the exploratory outcomes

##### 3.4.5.1 RPE

Four studies (28, 56, 62, 64) showed that H_2_ can significantly reduce RPE score as compared to the placebo, while another eight publications (nine studies) (23, 25, 26, 29, 34, 55, 58, 63) showed that H_2_ cannot significantly reduce RPE score (Table 1). The pooled ES of the RPE score was small and significant (SMD = −0.37, 95% CI −0.65 to −0.09, *p* = 0.009, Supplementary Figure S2), with moderate heterogeneity (*I*^2^ = 58.0%, *p* = 0.005). The funnel plot (Supplementary Figure S1F) and Egger’s test (*t* = −0.06, *p* = 0.955) indicated no publication bias.

The results of subgroup analyses revealed that strength training and repeated sprints yielded significant and large ESs (SMD = −1.41, 95% CI −2.32 to −0.50, *p* = 0.002 and SMD = −0.96, 95% CI −1.70 to −0.22, *p* = 0.011, respectively), while aerobic endurance exercise produced a significant and small ES (SMD = −0.33, 95% CI −0.59 to −0.07, *p* = 0.013). Conversely, anaerobic endurance exercise elicited small and non-significant ES (SMD = 0.29, 95% CI −0.25 to 0.83, *p* = 0.290). With regards to the source of H_2_, the ES was significantly large for HRG (SMD = −0.91, 95% CI −1.51 to −0.31, *p* = 0.003), while it was relatively small (SMD = −0.32, 95% CI −0.60 to −0.03, *p* = 0.029) for HRW.

##### 3.4.5.2 BLA

Five studies (34, 51, 53, 56, 64) showed that H_2_ can significantly improve BLA as compared to the placebo, while another eight publications (nine studies) (23, 25, 26, 29–31, 33, 55) showed opposite results that H_2_ cannot significantly improve BLA (Table 1). The pooled ES of BLA was small and significant (SMD = −0.37, 95% CI −0.60 to −0.15, *p* = 0.001, Supplementary Figure S3), with low heterogeneity (*I*^2^ = 22.0%, *p* = 0.215). The funnel plot (Supplementary Figure S1G) and Egger’s test (*t* = −3.44, *p* = 0.005) indicated that there was a potential risk of publication bias on these results, but the trim and fill method for sensitive analysis showed that the pooled ES (fixed: SMD = −0.349, *p* < 0.001; Random: SMD = −0.375, *p* = 0.001) was robust after filled meta-analysis.

The results of subgroup analyses revealed that aerobic endurance exercise yielded a significant and small ES (SMD = −0.38, 95% CI −0.67 to −0.08, *p* = 0.013), while anaerobic endurance exercise produced a non-significant and small ES (SMD = −0.67, 95% CI −1.72 to 0.38, *p* = 0.213); strength training elicited moderate and non-significant ES (SMD = −0.53, 95% CI −1.34 to 0.29, *p* = 0.206). Repeated sprints yielded a non-significant and small ES (SMD = −0.20, 95% CI −0.77 to 0.37, *p* = 0.496). With regards to the source of H_2_, the ES was significantly small for HRW (SMD = −0.42, 95% CI −0.68 to −0.15, *p* = 0.002), and the ES of HRG was small and not significant (SMD = −0.47, 95% CI −1.04 to 0.11, *p* = 0.111), while it was trivial (SMD = 0.00, 95% CI −0.65 to 0.65, *p* = 0.999) for HRC.

##### 3.4.5.3 HR_avg_

Two studies (22, 62) showed that H_2_ can significantly improve HR_avg_ during exercise as compared to the placebo, while another three studies (54, 55, 58) showed the opposite result that H_2_ cannot significantly improve HR_avg_ (Table 1). The pooled ES of HR_avg_ was not significant and small (SMD = −0.27, 95% CI −0.60 to 0.05, *p* = 0.094, Supplementary Figure S4) and without heterogeneity (*I*^2^ = 0%, *p* = 0.557). The funnel plot (Supplementary Figure S1H) and Egger’s test (*t* = 1.26, *p* = 0.296) indicated that there was no publication bias.

## 4 Discussion

To our knowledge, this is the first systematic review and meta-analysis exploring the effects of H_2_ supplementation on physical performance in healthy adults. The results suggest that H_2_ supplementation is promising for improving lower limb explosive power and reducing RPE and BLA clearance during vigorous exercise. However, it does not enhance endurance performance and muscle strength or decrease HR_avg_.

This meta-analysis suggests that administering H_2_ before or after exercise may serve as a potential strategy to effectively enhance lower limb explosive power in healthy adults. One potential mechanism underlying the effects of H_2_ on explosive power is that H_2_ can directly react with strong oxidants *in vivo* [e.g., hydroxyl radicals (•OH)] to modulate Ca^2+^ or mitochondrial ATP-dependent K^+^ channels, thus facilitating mitochondrial ATP production (20, 66–69). Additionally, H_2_ could reduce intracellular reactive oxygen species (ROS) levels and thus enhance muscle contractile function (27, 70). For example, a study conducted on soccer players demonstrated that administering three successive doses of 500 mL of HRW prior to high-intensity aerobic exercise increased the mean power frequency of skeletal muscles during subsequent strength tests (51). However, this finding that H_2_ promotes lower limb explosive power may be influenced by a small sample size (*n* = 92) or movement pattern. One example is that H_2_ significantly improved participants’ sprint performance compared to their vertical jump performance (25, 63). Therefore, more research is still needed to confirm this finding in the future. The result showed that H_2_ did not significantly improve muscle strength after aerobic endurance exercise. One possible reason is that intense aerobic exercise leads to a consumption of H_2_ in the body that does not continually provide benefits for subsequent muscle strength performance. One study (34) shows that 1,260 mL of HRW intake can increase the movement velocity of multiple lunges during resistance training. Therefore, more studies are needed in the future to clarify the effects of H_2_ supplementation on muscular strength performance in isolated resistance training. It has been observed that H_2_ supplementation cannot significantly improve aerobic and anaerobic endurance performance. Endurance performance depends on the multiple factors of human respiratory function, oxygen transport, and local muscle oxygen utilization during exercise (71, 72). Studies have shown that using H_2_ failed to significantly improve these critical factors (e.g., V̇O_2max_ and running economy) of endurance performance (23, 29, 31, 56, 57), thus leading to the insignificant benefits of H_2_ on this important function.

While H_2_ supplementation does not appear to enhance endurance performance or increase muscle strength, it does demonstrate favorable effects in reducing RPE, BLA levels, and HR_avg_ among individuals engaged in high-intensity exercise. H_2_ appears to be a neuroprotective agent that facilitates the restoration of neuronal oxidative damage by reducing oxidative stress and neuroinflammation (16, 73–75). H_2_ intake has also been reported to induce positive effects on exercise acidosis (56), thus modulating intracellular and extracellular buffering capacity during vigorous exercise (76). The decrease in BLA during exercise may be attributed to the fact that molecular H_2_ accelerates the transport of BLA to the liver for storage and oxidation, as well as increasing the utilization of lactate as a fuel by the muscles (56, 77). Subgroup analysis reveals that H_2_ supplementation reduces BLA concentration in aerobic endurance exercise, which is superior to other exercise types. The reduction in BLA response during aerobic endurance exercise may indicate that H_2_ supplementation enhances oxidative energy metabolism (28). Indeed, this finding may be unreliable due to the small number of studies on other exercise types. Therefore, future research should focus more on the effects of H_2_ supplementation on anaerobic endurance, muscular strength, and repeated sprint performance. Subgroup analyses reveal two important factors that likely contribute to the effects of H_2_ supplementation on RPE. First, we observed that the effects were greater in strength training and repeated sprints as compared to endurance exercise. The observed variations in RPE could be attributed to the disparities in the energy supply mechanisms across different types of exercises. It is plausible that H_2_ gas may exhibit a higher affinity toward the phosphagen system when compared to the oxidative and glycolytic systems (66). Second, inhalation of H_2_ gas (HRG) is superior to the ingestion of HRW in mitigating RPE. The observed discrepancy can be attributed to the fact that the respiratory absorption of molecular H_2_ is significantly more efficient and comprehensive in comparison to its digestive absorption in HRW. Nonetheless, given the restricted sample size, it is imperative to ensure further validation of the outcomes of the subgroup analysis.

## 5 Limitations

Five included studies with a small number of participants (*n* ≤ 10) (23, 27, 30, 52, 53) may lead to potential bias. Most studies to date focus on only younger and middle-aged men, and future studies are highly demanded to examine the benefits of H_2_ for women and those with older age. The current studies only investigated the effects of H_2_ supplementation for 1–14 days and future studies need to focus on the effects of longer supplementation periods. Some studies did not report or detect H_2_ concentrations, and the H_2_ dosing regimen was highly variable. The dose–response relationship between H_2_ and physical performance has not been established, which should be explored in the future to determine the most appropriate dosage and intervention protocol for H_2_ for enhancing physical performance.

## 6 Conclusion

In summary, this systematic review and meta-analysis suggest that short-term (<14 days) H_2_ supplementation protocols contribute to improved lower limb explosive power, fatigue relief, and BLA clearance but may not significantly improve aerobic endurance, anaerobic endurance, or muscular strength. Inhaling H_2_ shows promise as the optimal method for improving physical performance (i.e., lower limb explosive power) in healthy adults. Future studies with rigorous designs are needed to help obtain more definitive conclusions on the effects of H_2_ on lower limb explosive power and muscle strength in healthy adults.

## Data availability statement

The raw data supporting the conclusions of this article will be made available by the authors, without undue reservation.

## Author contributions

KZ: Data curation, Formal analysis, Methodology, Project administration, Software, Supervision, Writing – original draft, Writing – review & editing. ZS: Data curation, Formal analysis, Methodology, Software, Writing – original draft, Writing – review & editing. CY: Data curation, Formal analysis, Methodology, Writing – original draft. ZG: Conceptualization, Writing – original draft. YW: Formal analysis, Methodology, Software, Writing – original draft. DB: Conceptualization, Data curation, Formal analysis, Funding acquisition, Methodology, Resources, Supervision, Writing – original draft, Writing – review & editing. JZ: Conceptualization, Formal analysis, Methodology, Supervision, Writing – review & editing.
