# Supplementary material for: Can molecular hydrogen supplementation enhance physical performance in healthy adults? A systematic review and meta-analysis
Source: Front Nutr. 2024 Jun 5;11:1387657. doi: 10.3389/fnut.2024.1387657 (PMC11188335; doi:10.3389/fnut.2024.1387657)
Supplement: Supplementary file 1 [file Data_Sheet_1.docx]

Supplementary Material

**Can molecular hydrogen supplementation enhance physical performance in healthy adults? a systematic review and meta-analysis**

Kaixiang Zhou ^1, †^, Zhangyuting Shang ^2, †^, Chaoqun Yuan ^3^, Zhenxiang Guo ^4^, Yubo Wang ^5^, Dapeng Bao ^5, *^ and Junhong Zhou ^6^

^1^ College of Physical Education and Health Science, Chongqing Normal University, Chongqing, China;

^2^ College of Physical Education and Health Management, Chongqing University of Education, Chongqing, China;

^3^ College of Sports and Health, Chengdu University of Traditional Chinese Medicine, Chengdu, Sichuan, China;

^4^ Sports Coaching College, Beijing Sport University, Bejing, China;

^5^ China Institute of Sport and Health Science, Beijing Sport University, Beijing, China;

^6^ Hebrew SeniorLife Hinda and Arthur Marcus Institute for Aging Research, Harvard Medical School, Boston, Massachusetts, USA.

*** Correspondence:** Dapeng Bao, PhD, China Institute of Sport and Health Science, Beijing Sport University, 48 Information Road, Haidian District, Beijing, China; e-mail address: baodp@bsu.edu.cn.

# Supplementary Figures and Tables

## Figure S1 Funnel plots.

## Figure S2 Forest plot of the effects of H2 supplementation on RPE.

## Figure S3 Forest plot of the effects of H2 supplementation on blood lactate.

## Figure S4 Forest plot of the effects of H2 supplementation on HR_avg_.

## Table S1 Search Strategy

## Table S2 The quality of the evidence (GRADE)


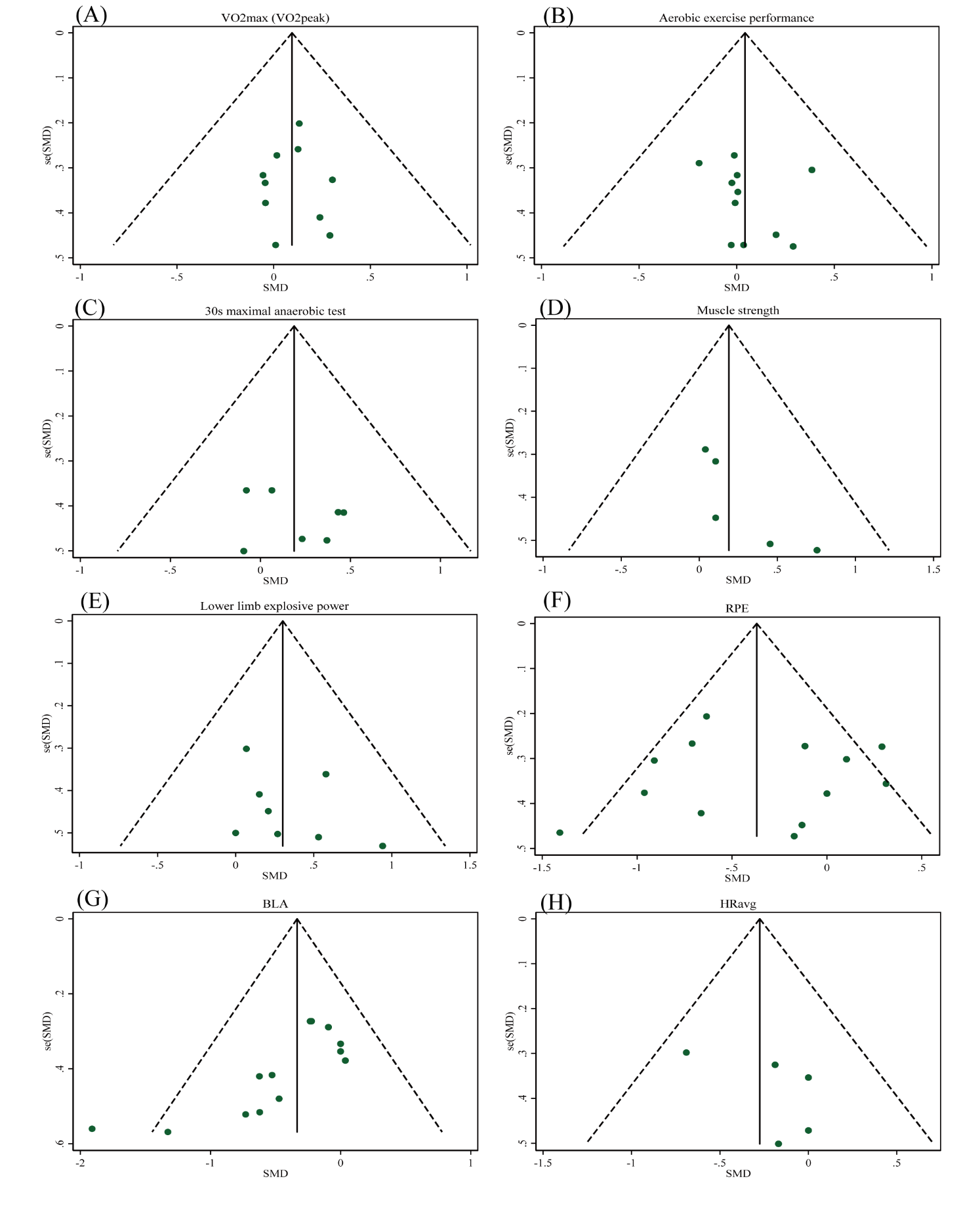


**Figure S1 Funnel plots.**


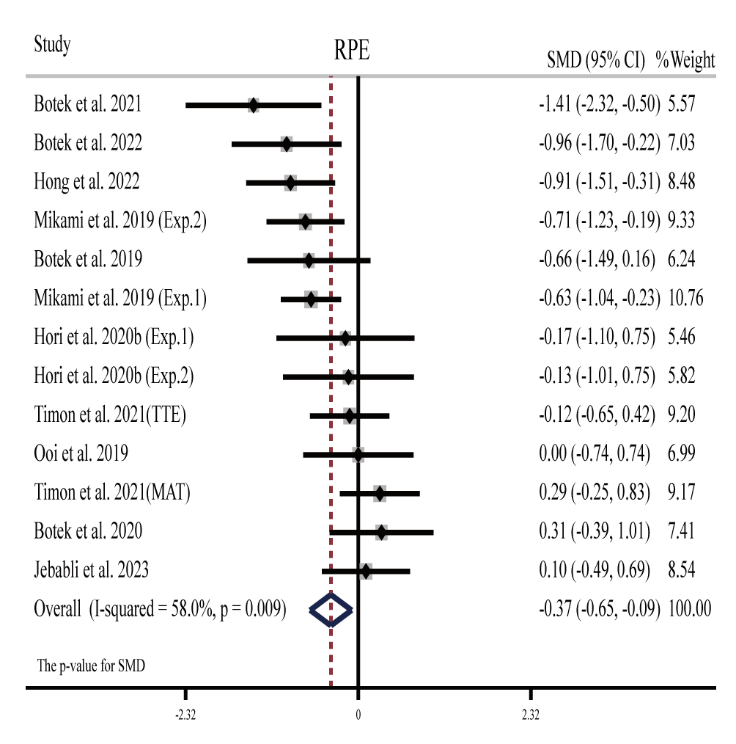


**Figure S2 Forest plot of the effects of H_2_ supplementation on RPE.**Exp.1, Experiment 1; Exp.2, Experiment 2; TTE, Time-to-exhaustion; MAT, 30s Maximal anaerobic test


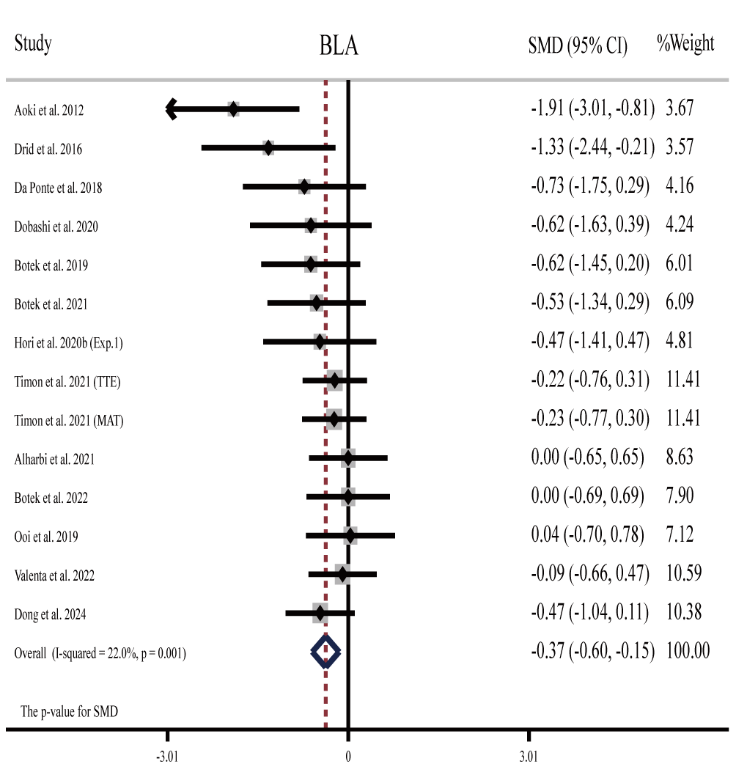


**Figure S3 Forest plot of the effects of H_2_ supplementation on blood lactate.** Exp.1, Experiment 1; Exp.2, Experiment 2; TTE, Time-to-exhaustion; MAT, 30s Maximal anaerobic test


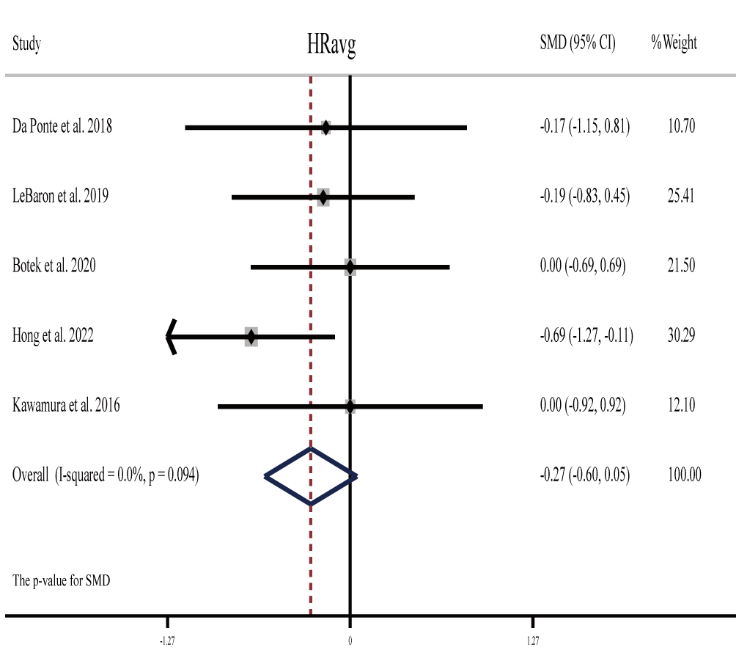


**Figure S4 Forest plot of the effects of H_2_ supplementation on HR_avg_.**

**Table S1: Search Strategy**

| Process | Keywords |
| --- | --- |
| # 1 | "molecular hydrogen" OR "hydrogen rich water" OR "hydrogen-rich water" OR "hydrogen rich saline" OR "hydrogen-rich saline" OR "H_2_-dissolved water" OR "H_2_-infused water" OR "hydrogen gas" OR "hydrogen inhalation" OR "hydrogen bathing" OR "hydrogen-rich calcium powder" |
| # 2 | "sports performance" OR "athletic performance" OR "exercise performance" OR "physical performance" OR "physical fitness testing" OR "physical exercise" OR "exercise capacity" OR "aerobic performance" OR "endurance performance" OR "aerobic fitness" OR "aerobic capacity" OR "anaerobic performance" OR "anaerobic endurance" OR "anaerobic capacity" OR "Wingate anaerobic test" OR "blood lactate" OR "blood lactic acid" OR "heart rate" OR "maximal oxygen uptake" OR "running economy" OR "running to exhaustion" OR "bicycle ergometry test" OR "incremental cycling exercise test" OR "incremental treadmill test" OR "step tests" OR "field testing" OR "intermittent exercise" OR sprint OR "countermovement jump" OR "repeated sprints" OR "swimming" OR "rowing" OR "strength training" OR "resistance training" OR "muscle endurance" OR "maximal voluntary isometric strength" |
| # 3 | # 1 AND # 2 |

**Table S2: The quality of the evidence (GRADE)**

| **Quality assessment** | | | | | | | **No of participants** | |  | **Quality** | **Importance** |  |
| --- | --- | --- | --- | --- | --- | --- | --- | --- | --- | --- | --- | --- |
|  |  |  |  |  |  |  |  |  |  |  |  |  |
| **No of studies** | **Design** | **Risk of bias** | **Inconsistency** | **Indirectness** | **Imprecision** | **Other considerations** | **H2** | **Placebo** | **Absolute** |  |  |  |
|  |  |  |  |  |  |  |  |  |  |  |  |  |
| **V̇O2max (Better indicated by lower values)** | | | | | | | | | | | |  |
| 10 | randomised trials | no serious risk of bias | no serious inconsistency | no serious indirectness | no serious imprecision | none | 211 | 206 | SMD 0.09 higher (0.1 lower to 0.29 higher) | ⊕⊕⊕⊕ | CRITICAL |  |
|  |  |  |  |  |  |  |  |  |  | HIGH |  |  |
| **Aerobic exercise performance (Better indicated by lower values)** | | | | | | | | | | | |  |
| 11 | randomised trials | no serious risk of bias | no serious inconsistency | no serious indirectness | no serious imprecision | reporting bias^1^ | 178 | 178 | SMD 0.04 higher (0.17 lower to 0.25 higher) | ⊕⊕⊕O | CRITICAL |  |
|  |  |  |  |  |  |  |  |  |  | MODERATE |  |  |
| **30s maximal anaerobic performance (Better indicated by lower values)** | | | | | | | | | | | |  |
| 7 | randomised trials | no serious risk of bias | no serious inconsistency | no serious indirectness | no serious imprecision | none | 80 | 80 | SMD 0.19 higher (0.12 lower to 0.5 higher) | ⊕⊕⊕⊕ | CRITICAL |  |
|  |  |  |  |  |  |  |  |  |  | HIGH |  |  |
| **Lower limb explosive power (Better indicated by lower values)** | | | | | | | | | | | |  |
| 10 | randomised trials | no serious risk of bias | no serious inconsistency | no serious indirectness | no serious imprecision | none | 128 | 128 | SMD 0.30 higher (0.05 to 0.55 higher) | ⊕⊕⊕⊕ | CRITICAL |  |
|  |  |  |  |  |  |  |  |  |  | HIGH |  |  |
| **Muscle strength (Better indicated by lower values)** | | | | | | | | | | | |  |
| 4 | randomised trials | no serious risk of bias | no serious inconsistency | no serious indirectness | no serious imprecision | none | 50 | 50 | SMD 0.19 higher (0.14 lower to 0.52 higher) | ⊕⊕⊕⊕ | CRITICAL |  |
|  |  |  |  |  |  |  |  |  |  | HIGH |  |  |
| **RPE (Better indicated by lower values)** | | | | | | | | | | | |  |
| 13 | randomised trials | no serious risk of bias | serious^2^ | no serious indirectness | no serious imprecision | none | 271 | 266 | SMD 0.37 lower (0.65 to 0.09 lower) | ⊕⊕⊕O | IMPORTANT |  |
|  |  |  |  |  |  |  |  |  |  | MODERATE |  |  |
| **Blood Lactate (Better indicated by lower values)** | | | | | | | | | | | |  |
| 14 | randomised trials | no serious risk of bias | no serious inconsistency | no serious indirectness | no serious imprecision | reporting bias^3^ | 217 | 217 | SMD 0.37 lower (0.60 to 0.15 lower) | ⊕⊕⊕O | IMPORTANT |  |
|  |  |  |  |  |  |  |  |  |  | MODERATE |  |  |
| **HR_avg_ (Better indicated by lower values)** | | | | | | | | | | | |  |
| 5 | randomised trials | no serious risk of bias | no serious inconsistency | no serious indirectness | no serious imprecision | none | 76 | 76 | SMD 0.27 lower (0.6 lower to 0.05 higher) | ⊕⊕⊕⊕ | IMPORTANT |  |
|  |  |  |  |  |  |  |  |  |  | HIGH |  |  |

^1^ The funnel plot and Egger’s test (t=2.44, p=0.041) indicated that there was a potential risk of publication bias on this result.
^2^ *I*^2^=57.5%
^3^ The funnel plot and Egger’s test (t=-3.84, p=0.003) indicated that there was a potential risk of publication bias on this result.
